# Antibody–Drug Conjugates Targeting CD30 in T-Cell Lymphomas: Clinical Progression and Mechanism

**DOI:** 10.3390/cancers17030496

**Published:** 2025-02-02

**Authors:** Yi Jiang, Sai Dong, Yang Wang

**Affiliations:** 1 Department of Dermatology and Venereology, Peking University First Hospital, Beijing 100034, China; doctorjiang1999@163.com (Y.J.); ds123456789@stu.pku.edu.cn (S.D.); 2 National Clinical Research Center for Skin and Immune Diseases, Beijing 100034, China; 3 NMPA Key Laboratory for Quality Control and Evaluation of Cosmetics, Beijing 100034, China; 4 The Second Clinical Medical School, Peking University, Beijing 100044, China; 5 Peking-Tsinghua Center for Life Sciences, Peking University, Beijing 100871, China

**Keywords:** T-cell lymphomas, antibody–drug conjugate, CD30, brentuximab vedotin, tumor microenvironment, drug resistance, anticancer combination therapy

## Abstract

CD30 is overexpressed in many T-cell lymphoma (TCL) entities, including subsets of peripheral T-cell lymphomas (PTCL) and cutaneous T-cell lymphomas (CTCL). The antibody–drug conjugate brentuximab vedotin (BV), targeting CD30-positive cells, has been approved for the treatment of relapsed or refractory (R/R) systemic anaplastic large cell lymphoma (sALCL), and primary cutaneous anaplastic large cell lymphoma or mycosis fungoides in patients who have received previous systemic therapy. However, many patients still experience disease progression after BV monotherapy. Extensive efforts have been dedicated to investigating effective combinations of BV. A phase III clinical study demonstrated that the combination of BV with cyclophosphamide, doxorubicin, and prednisone (CHP) is superior to cyclophosphamide, doxorubicin, vincristine, and prednisone (CHOP) for CD30-positive PTCL. This study led to the approval of BV with CHP as the first-line therapy for CD30-positive PTCL (sALCL in Europe). We summarize the encouraging combination applications of BV in this review. Ongoing studies on combination therapies of BV are also listed, highlighting potential directions for the future application of BV. We focus on dissecting the underlying mechanisms of BV, discussing its effects on both tumor cells and the tumor microenvironment. Exploring resistance mechanisms in TCL provide valuable insights for optimizing BV-based therapies in the future.

## Simple Summary

CD30 is overexpressed in many T-cell lymphomas. The antibody–drug conjugate brentuximab vedotin (BV) targets CD30-positive cells and is approved for the treatment of several lymphoma types. However, the development of resistance limits the long-term efficacy of BV. This review comprehensively summarizes the key clinical trials of BV as monotherapy and in combination with other therapies for T-cell lymphomas. Ongoing studies exploring BV combination therapies are listed, highlighting potential directions for its future application. To improve the understanding and efficacy of BV, we discuss the action mechanisms of BV in tumor cells and the tumor microenvironment. The mechanisms of resistance are also discussed to offer new insights into overcoming them, laying the groundwork for optimizing BV treatment strategies in the future.

## 1. Introduction

T-cell lymphomas (TCL) are a heterogeneous group of mature T-derived malignancies, categorized broadly as peripheral T-cell lymphoma (PTCL) and cutaneous T-cell lymphoma (CTCL). PTCL mainly consists of PTCL-not otherwise specified, systemic anaplastic large cell lymphoma (sALCL), and angioimmunoblastic T-cell lymphoma [1]. Most PTCL subtypes are aggressive and chemotherapy-resistant. Despite development in novel therapies, the 5-year overall survival (OS) rates are as low as 30–40% [2]. Around 70% of patients develop relapsed or refractory (R/R) disease after the first-line therapy, and treatment options are limited [3]. As for CTCL, the most common subtypes are mycosis fungoides (MF), Sézary syndrome (SS), and CD30-positive lymphoproliferative disorders, which include primary cutaneous anaplastic large cell lymphoma (pcALCL) and lymphomatoid papulosis (LyP) [4]. Most patients undergo slow progression disease over the years. However, advanced-stage CTCL requires systemic treatment and has a poor prognosis with increased mortality [5,6].

The investigation of more effective treatments for TCL is very active. Recently, novel agents such as brentuximab vedotin (BV) and immune checkpoint inhibitors have shown high efficacy and tolerability in the treatment of patients with R/R TCL or those previously treated [7,8,9,10,11]. BV is an antibody–drug conjugate (ADC) that links the monoclonal antibody (mAb, cAC10), targeting CD30, to the antitubulin drug monomethylauristatin E (MMAE) via a protease-cleavable linker. After binding to CD30 on tumor cells, BV releases MMAE upon internalization, which induces cell cycle arrest and therefore apoptosis [12,13,14,15,16,17,18]. Based on its effectiveness in treating TCL with manageable adverse effects, BV has been approved as monotherapy for R/R sALCL, previously treated pcALCL, and MF. In Europe, the combination of BV with CHP serves as a front-line therapy for previously untreated sALCL patients, while in the USA, this combination is additionally applied to other CD30-positive PTCL patients [9,19,20]. Given the clinical significance and extensive real-world data availability of BV, it serves as the primary focus for a comprehensive understanding of CD30-targeting ADC compared to other CD30-targeting ADCs that are still under preclinical or clinical phase I studies [21,22,23]. Despite the promising effects of BV, many patients still suffer progressive disease after treatment and eventually develop resistance, while the underlying mechanisms are not fully understood [3,24,25]. In this review, we summarize the successful applications of BV as monotherapy and in combination with other therapies for TCL. We focus on discussing its mechanisms of action and resistance, aiming to provide insights to enhance BV-based therapies in the future.

## 2. Efficacy of BV in the Treatment of TCL

BV is an ADC that targets CD30-positive tumor cells. CD30, a member of the tumor necrosis factor receptor superfamily, has limited expression in a subset of activated lymphocytes and plays an important role in immune response and regulation [26,27]. Additionally, CD30 is preferentially expressed on tumor cells across various lymphoma subtypes, and its overexpression characterizes classic Hodgkin lymphoma (cHL) and sALCL [28,29,30]. Variable levels of CD30 expression have also been observed in other TCLs, including pcALCL and transformed MF (see Table 1). Despite the diagnosis and prognostic evaluation, the downstream effects of CD30 in tumors are not fully understood, while the overall effects depend on the cell type and its differentiation state [31,32,33,34,35]. Studies have revealed that CD30 induces survival signals and promotes proliferation through NF-kB and mitogen-activated protein kinase signaling pathways [26,36,37,38,39]. Nonetheless, the relatively specific overexpression in tumor cells makes CD30 a preferable target for ADC therapy.

In early studies, BV showed strong and selective antitumor activity in vitro and in mouse models [51,52,53]. Encouraged by preclinical data, a phase I trial established an intravenous dose of 1.8 mg/kg every three weeks as the maximum tolerated dose of BV, with dose-limiting toxicities observed in patients with R/R CD30-positive lymphomas [54]. Based on that, numerous clinical trials in TCL were conducted. A multicenter phase II clinical trial enrolled 58 sALCL patients with recurrent disease after at least one prior therapy. Among them, 50 patients (86%) achieved objective response (OR), including 33 complete remission (CR) and 17 partial remission (PR) patients [55]. No disease progressions were observed beyond 40 months among all patients. Peripheral neuropathy occurred in 33 patients, but the majority experienced resolution or improvement by their last assessment [24]. Another phase II multicenter study further established the activity of single-agent BV in R/R PTCL. Of 34 evaluable patients, the OR rate was 41%, including eight with CR and six with PR. The median duration of response was 7.6 months. Moreover, they found no correlation between CD30 expression levels and treatment response [8].

The efficacy and safety of BV in CTCL were evaluated in a phase II study involving 48 patients with LyP/pcALCL and MF/SS [56]. The patients had an OR rate of 73% and CR rate of 35%, with responses seen in 54% of patients with MF and 100% of patients with LyP/pcALCL. Responses in MF/SS were also independent of CD30 expressions, and the OR rate was 50% in patients with low CD30 expression (<10%). However, another phase II study found that the median CD30 expression level was higher in responders compared to non-responders [57]. Both studies showed manageable adverse events. An international randomized phase III study (ALCANZA) was conducted to compare BV with physician’s choice of standard therapy (methotrexate or bexarotene) in previously treated CD30-positive CTCL patients, including 97 patients with MF and 31 patients with pcALCL [58]. This study revealed more favorable OR rate lasting at least 4 months (56.3% vs. 12.5%) and a longer median progression-free survival (PFS, 16.7 months vs. 3.5 months) of patients in the BV group compared to the physician’s choice group. Similarly, responses to BV were seen across a full range of CD30 expression levels. Adverse events were comparable between both groups. Based on this, BV was approved by the Food and Drug Administration (FDA) and the European Commission for the treatment of CD30-positive MF after prior systemic therapy. Clinical trials have also been conducted to manage the efficacy of BV in R/R PTCL patients with low CD30 expression (<10%) (NCT02588651) and to optimize the dose of BV for CTCL treatment (NCT03587844).

## 3. Studies on the Combination Therapy of BV for TCL

The potential of BV in combination therapies has been investigated to optimize its efficacy (see Table 2). Most importantly, the randomized, placebo-controlled phase III trial ECHELON-2 confirmed that front-line treatment with BV and cyclophosphamide, doxorubicin, and prednisone (CHP) was superior to cyclophosphamide, doxorubicin, vincristine, and prednisone (CHOP) for patients with CD30-positive PTCL [7]. BV in combination with CHP showed a significant improvement in median PFS (48.2 months vs. 20.8 months, HR = 0.71, *p* = 0.0010) and OS (median not reached, HR = 0.66, *p* = 0.0244), with a manageable safety profile. The rates and severity of adverse events, including febrile neutropenia and peripheral neuropathy, were similar between both groups. This pivotal study led to the FDA approval of BV in combination with chemotherapy for CD30-positive PTCL patients. Subsequent studies have further confirmed the promising efficacy of BV combined with chemotherapy [59,60].

BV has also demonstrated satisfactory results when combined with other treatment modalities. For instance, the combination of BV with ultra-hypofractionated low-dose total skin electron beam therapy led to rapid responses in three patients with tumor-stage MF (stage IIB) [61]. The combination of BV with nivolumab, an anti-PD-1 mAb, was tolerable and showed an OR rate of 44% after four cycles in patients with BV-refractory CD30-positive lymphoma [62]. The addition of skin-directed therapies after progression under BV monotherapy could stabilize the disease’s continuous advancement or even lead to PR in CTCL patients [63]. Additionally, a preclinical study revealed that chidamide, a novel histone deacetylase inhibitor (HDACi), exhibited a strong synergistic effect with BV in TCL cell lines and xenograft mice models [64]. However, further validation in clinical settings is needed. Another genome-wide screening study found that genes functioning as endogenous inhibitors of the anaphase-promoting complex, which enhanced sensitivity to BV in ALCL cells, whose inhibitors had a synergistic effect with BV in cell lines and peripheral blood mononuclear cells from a PTCL patient [65]. However, further validation in patients is required. An increasing number of studies are being conducted to evaluate the efficacy of BV in combination with other drugs, including previously reported treatments such as chemotherapy, HDACi, and immune checkpoint inhibitor, as well as new drugs like CCR4 mAb (see Table 2).

**Table 2:** Clinical studies of BV in combination treatment for PTCL and CTCL.

Designation	Phase	Patient	CD30 Criteria	Treatment	Key Findings/Status	AEs	Citation
NCT01309789	I	Treatment-naïve PTCL (*n* = 38)	≥1%	BV→CHOP or BV + CHP→BV maintenance	ORR 85% with CRR 62% for BV→CHOP ORR 100% with CRR 88% for BV + CHP 5-year PFS 52% and OS 80% for BV + CHP	77% in BV→ CHOP treatment and 69% in BV →BV+CHOP treatment. 18 of 19 patients improved or resolute of PN, including 9 complete resolutions	[66,67]
NCT01777152	III	Treatment-naïve PTCL (*n* = 452)	≥10%	Randomized to BV-CHP vs. CHOP	ORR 83% with CRR 68% for BV-CHP ORR 72% with CRR 56% for CHOP mPFS 48.2 months for BV-CHP vs. 20.8 months for CHOP 5-year OS 75.1% for BV-CHP vs. 61.0% for CHOP, HR = 0.72.	Similar between groups PN 52% in BV+CHP and 55% in CHOP	[7,68]
NCT03496779	II	R/R PTCL (*n* = 71)	≥5%	BV + Gemcitabin →BV maintenance	ORR 47.9% with CRR 19.7% and PRR 28.2%, DOR 12.8 months	PN 11% Grade ≥3 AEs 81.7%	[69]
NCT03217643	II	Treatment-naïve CD30-positive EATL type 1 (*n* = 14)	≥10%	BV + CHP→ASCT	ORR 79% with CRR 64% No relapse, 2-year PFS 63% and OS 68%	Consistent with known safety profiles of BV-CHP	[70]
NCT02616965	I	MF or LyP/pcALCL required systematic therapy (*n* = 15)	Not required	BV + Romidepsin	ORR 64% (7/11) with 1 CR patient. median mSWAT decrease of 57% mPFS 5.5 months, mOS not reached with 1-year OS 81%	Nausea 67%, PN 33%; Grade 3 AEs (*n* = 3) resolved spontaneously	[71]
NCT03246750	I/II	Treatment-naïve ENKTL (*n* = 34)	Not reported	BV + MAD	CRR 66.7%, PRR 16.7% in 30 patients, 16.7% progressed ORR and CRR in localized and advanced diseases were 89.5%/78.9% and 72.7%/45.5%	Anemia 10%, PN 3.7%; Grade ≥ 3 AEs 12.6%. 21 SAEs but manageable and completely resolved upon follow-up.	[72]
NCT01703949	II	BV-refractory cHL (*n* = 14), ALK^-^ ALCL (*n* = 2), CD30^+^ MF (*n* = 3)	Not reported	BV + Nivolumab	ORR 44% with CRR 19% after 4 cycles. ORR and CRR 100% and 50% in ALK- ALCL, 100% and 100% in MF. Median time to progression and next therapy 4.5 and 5.0 months No correlation between correlative PD-1 levels and response.	63% and 5% grade 1 and 2 PN at the end of treatment.	[62]
NCT03113500	II	Treatment-naïve PTCL (*n* = 48)	≥1%	BV + CHEP	ORR 91% (43/47) with CRR 79% (37/47) and PRR 13% (6/47)	PN 56%, fatigue 71%, nausea 58%	[60]
NCT04569032 *	II	non-sALCL PTCL (*n* = 70)	<10%	BV + CHP	ORR 77% with CRR 65% among 66 patients	Grade ≥3 AEs 61%, 7 patients discontinued treatment due to AEs. 19 patients had BV-related SAEs, including 2 deaths	[59]
NCT05673785	II	Treatment-naïve CD30-posive PTCL	Not reported	BV + CHP	Recruiting		
NCT05414500	I	MF/SS with prior systemic therapy	>1%	BV + Mogamulizumab	Recruiting	NA	NA
NCT05357794	II	R/R MF	>1%	BV + ULD-TSEBT	Recruiting	NA	NA
NCT05316246	II	R/R NK/TCL	≥10%	BV + Tislelizumab	Unknown status	NA	NA
NCT05313243	II	R/R TCL	>1%	BV + Pembrolizumab → Pembrolizumab	Recruiting	NA	NA
NCT03719105	I	Advanced PTCL (non-ALCL or non-NK/TCL, cohort 2)	Not reported	Pralatrexate (cycles 1, 2, 4, 6) and BV (cycles 3, 5) + CHP → ASCT for responders	Recruiting	NA	NA
NCT03409432	II	R/R TCL	Not required	BV + Lenalidomide	Active, not recruiting	NA	NA
NCT03264131	II	ATLL	Not required	BV + CHEP	Active, not recruiting	NA	NA

* A new cohort is ongoing. PTCL, peripheral T-cell lymphoma; CTCL, cutaneous T-cell lymphoma; (S)AEs, (severe) adverse effects; BV, brentuximab vedotin; CH(O/E)P, cyclophosphamide, doxorubicin, (vincristine/etoposide), prednisone; ORR, objective response rate; CRR, complete response rate; (m)OS, (median) overall survival; PN, peripheral neuropathy; (m)PFS, (median) progression-free survival; HR, hazard ratio; R/R, relapsed or refractory; PRR, partial response rate; DOR, duration of response; EATL, enteropathy-associated T-cell lymphoma; ASCT, autologous stem cell transplantation; MF, mycosis fungoides; LyP, lymphomatoid papulosis; (pc/s)ALCL, (primary cutaneous/systematic) anaplastic large-cell lymphoma; mSWAT, modified severity-weighted assessment tool; ENKTL extra-nodal natural killer/T-cell lymphoma; MAD, methotrexate, L-asparaginase, dexamethasone; cHL, classical Hodgkin lymphoma; SS, Sézary syndrome; NA, not applicable; ULD-TSEBT, ultra-low-dose total skin electron beam therapy; NK/TCL, natural killer/T-cell lymphoma; ATLL, adult T-cell lymphoma.

## 4. Mechanisms of BV Treatment for TCL

Despite the successful application of BV in TCL, there are still some controversies and unsettled issues, such as the association between CD30 expression and treatment efficacy, and the presence of non-responders. Studies are required to address these questions and provide more insights into the mechanisms underlying BV treatment [10,24,73].

**Figure 1:**
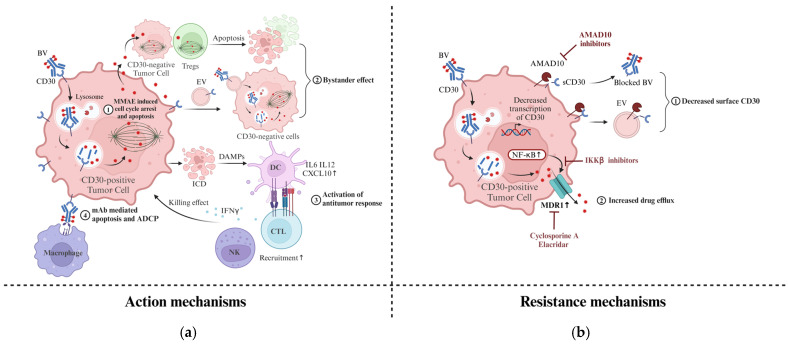
Action and resistance mechanisms of BV. (**a**) Action mechanisms of BV. After binding to CD30 on the surface of tumor cells, BV is endocytosed and transported to lysosomes, where the linker is degraded, releasing MMAE into the cytoplasm. (1) Free MMAE binds to microtubule to arrest mitosis in the M phase and induces apoptosis; (2) Free MMAE diffuses or is transported by EVs into bystander cells in the TME and exerts toxicity; (3) ICD induced by MMAE activates antitumor responses in TME; (4) The mAb of BV induces direct apoptosis of tumor cells and ADCP by macrophage. (**b**) Mechanisms of resistance to BV. (1) Decreased transcription of CD30, cleavage by ADAM10 on the cell surface and EVs downregulates the cell surface CD30 levels, which reduces the available targets for BV; (2) Activation of the NF-κB pathway results in increased expression of MDR1, which enhances drug efflux. BV, brentuximab vedotin; MMAE, monomethylauristatin E; EV, extracellular vesicles; ICD, immunogenic cell death; DAMPs, damage-associated molecular patterns; DC, dendritic cell; CTL, cytotoxic T lymphocyte; TME, tumor microenvironment; NK, natural killer cell; ADCP, antibody-dependent cellular phagocytosis; mAb, monoclonal antibody; sCD30, soluble CD30; ADAM10, a disintegrin and metalloproteinase 10; MDR1, multidrug resistance protein 1; Image created with BioRender.com.

After binding to the CD30 ligand on the surface of tumor cells, BV is rapidly internalized and transported to lysosomes. The valine–citrulline dipeptide linker is then degraded by cathepsin B, and MMAE is released to bind to tubulin and disrupts microtubule formation, which results in cell cycle arrest in the M phase and following apoptosis [14]. Many studies have confirmed that BV can induce the death of CD30-positive tumor cells in vitro and in mouse models [52,74,75]. Additional actions of BV have been uncovered, including the bystander effect of MMAE, activation of antitumor responses via immunogenic cell death (ICD), mAb-mediated apoptosis, and antibody-dependent cellular phagocytosis (ADCP) by macrophage (see Figure 1a).

### 4.1. MMAE-Induced Cell Apoptosis

MMAE is a synthetic analog of the natural product dolastatin 10. It binds to tubulins and effectively blocks tubulin polymerization and microtubule protein-dependent GTP hydrolysis [76]. Due to its higher stability, potency, and water solubility compared to dolastatin 10, MMAE has become a priority payload used in ADCs [77].

Upon internalization into the CD30-positive cells, unmodified drug MMAE is released from BV through proteolytic cleavage of the dipeptide linker and binds to tubulin. This induces cell cycle arrest and following apoptosis. The released MMAE is relatively more permeable across the cell membrane and can diffuse into the neighboring tumor microenvironment (TME). It forms a concentration gradient around target cells and causes additional cell killing in nearby CD30-negative cells, which is termed the ‘bystander effect’ of ADCs [16,78,79]. Tumor cells are highly sensitive to free MMAE in vitro, regardless of CD30 expression levels [78]. The potency of the bystander effect relies on the antigen-positive cells since it increases with the increasing ratio of target antigen-positive to antigen-negative cells and declines over time as the ADC works [80]. This overcomes the heterogeneity in CD30 expression among malignant cells and enhances the overall efficacy of BV [52]. However, close bystander cells might nonspecifically take up the cytotoxic MMAE, and no study has clarified how it influences the nearby antitumor cells yet. Notably, BV has also been found to be effective in PTCL and B-cell lymphomas with undetectable CD30 via immunohistochemistry (IHC) [81,82]. Potential mechanisms include the release of MMAE from BV by extracellular enzymes, false negatives in CD30 detection by IHC, and the heterogeneous expression of CD30 in tumor samples [81,83]. However, further validations are needed to confirm these hypotheses. In addition, studies have shown that extracellular vesicles (EVs) from CD30-positive cells contain CD30 and can bind to BV. These EVs are internalized by bystander cells and exert toxic activity of BV similar to that in CD30-positive cells [84,85].

### 4.2. Activation of Antitumor Immune Response in TME

ICD represents a unique variant of regulated cell death driven by stress or certain antitumor treatments [17,86,87]. It evokes an adaptive immune response against antigens from dying cells [88]. Studies have shown that tumor-associated antigens alone are not sufficient to drive the maturation of antigen-presenting cells, mainly dendritic cells (DCs). Damage-associated molecular patterns released during ICD strongly promote DC maturation by binding to specific pattern recognition receptors and ultimately activate both the innate and adaptive immune responses in support of treatment efficacy [88,89,90].

Heiser et al. [17] found that BV, as well as MMAE, were able to induce classical ICD in cHL cell lines by interfering with normal endoplasmic reticulum (ER) functions and triggering ER stress. ER stress is characterized by (1) the expression of calreticulin (CRT) on the cell surface, (2) the secretion of ATP, and (3) the passive release of high-mobility group protein B1. Both co-culture and xenograft mouse models demonstrated that BV- or MMAE-treated tumor cells significantly induced DC maturation, promoting the secretion of pro-inflammatory factors from DCs (IL1β, IL6, IL12, MIP-1α, and CXCL10) and CD8^+^ effector T cells (IFNγ and TNFα) [17,18]. MMAE, or guanine nucleotide exchange factor-H1 released upon microtubule destabilization, enhanced cross-presentation of tumor antigens to CD8^+^ T cells [91]. These, in turn, augmented the antigen-specific immune responses within the TME. Additionally, BV promoted the formation of immune memory and protected mice from tumor re-attack [17]. Depletion of DCs, CD8^+^ T cells, or neutralization of IFNγ with antibodies significantly reduced the antitumor effects of dolastatin 10 in mice [18]. BV and dolastatin 10 could also promote the recruitment of CD8^+^ T cells and natural killer (NK) cells to the TME and decrease the proportion of regulatory T cells (Tregs) [17,18]. The inhibitory effect of BV on Tregs may originate from BV-induced ICD or from the bystander effect exerted by MMAE [52]. However, no study has yet clearly revealed this mechanism and failed to elucidate why BV prefers to suppress Tregs rather than effector T cells. These queries may become important directions for future studies. In combination with anti-PD-1, the antitumor effect of BV was significantly enhanced in mouse models [17,18]. Clinical studies have also demonstrated the efficacy and safety of BV in combination with nivolumab (anti-PD-1) or ipilimumab (anti-CTLA-4) for the treatment of R/R TCL and cHL [62,92,93].

### 4.3. CD30 mAb–Mediated Apoptosis and ADCP

The antitumor mechanism of BV may also originate from its ability to mimic the CD30 ligand. The chimeric mAb cAC10, also known as SGN-30, possesses a variable region of a mouse antihuman CD30 antibody and a constant region of human α1 heavy and κ light chains. It retains the binding, in vitro growth-inhibitory, and ADCP activities of its parent antibody [75,94,95,96]. Early studies demonstrated that SGN-30 induced cellular growth arrest and DNA fragmentation in cHL and ATLL cell lines. It promoted receptor cross-linking and multimerization, potentially activating the classical apoptotic pathway induced by p21 or c-myc [95,96,97]. Additionally, SGN-30 combined with the chemotherapies bleomycin, Ara-C, and etoposide resulted in synergistic effects in a mouse xenograft model [94]. cHL cell lines pretreated with SGN-30 were more sensitive to classical chemotherapies, including doxorubicin, bleomycin, vincristine, dacarbazine, ifosfamide, carboplatin, and etoposide [96,98]. A study revealed that the antitumor effect of SGN-30 was significantly reduced in a macrophage-deficient mouse model, whereas the ablation of NK cells had no impact, suggesting that such effect mainly derives from macrophage-mediated ADCP rather than complement-dependent cytotoxicity or antibody-dependent cytotoxicity [75]. Based on the promising results in preclinical studies, phase I and II trials of SGN-30 in CD30-positive R/R HL and CD30-positive non-HL were undertaken [99,100,101]. However, the clinical outcomes were not satisfying and did not seem to offer significant promise for the use of ‘naked’ anti-CD30 mAb in practice.

## 5. Mechanisms and Solutions of Resistance to BV Treatment

Despite the promising OR rate, many patients still suffer from progressive disease and have poor outcomes after BV monotherapy [10,24,73,102,103,104]. The mechanisms of resistance are not fully understood. The potential mechanisms of resistance to ADCs have been summarized in previous studies, including (1) alterations in target antigen such as decreased expression levels and accessibility; (2) defects in internalization and trafficking pathways; (3) impaired lysosomal function; (4) direct resistance to payload drugs, such as increased drug efflux pumps; (5) apoptotic dysregulations [105,106,107]. A few of these mechanisms have been reported in BV (see Figure 1b).

### 5.1. Decreased CD30 on Cell Surface

BV resistance may be associated with decreased CD30 expression on the tumor cell surface. Previous studies have successively reported reduced or absent CD30 expression after treatment with BV in TCL patients [103,104,108,109]. A higher cumulative BV dose and low initial CD30 expression in tumor cells from the initial biopsy might be predictors of a loss or decrease in CD30 expression in ALCL patients. Chen et al. [110] constructed a BV-resistant ALCL cell line and observed significant CD30 downregulation compared with the parental cell line. However, they demonstrated that the sorted CD30-positive subpopulation was still resistant to BV despite significant CD30 expression. The percentage of CD30-positive cells in the population did not correlate with the degree of BV resistance. Samples from patients who relapsed were resistant to BV persistently expressed CD30 by IHC [110]. BV was found effective for PTCL regardless of CD30 expression status in some clinical studies, but less effective in CTCL patients with CD30 expression <5% [57,68,81]. A recent study found that the knockout of CD30 decreased the sensitivity of CD30-positve ALCL cells to BV by almost 100% [65]. The contradictory results may result from the following: (1) differences in assays for detecting and assessing CD30 expression levels, such as whether CD30 is expressed on the cell surface or intracellularly (or both); (2) differences in the baseline characteristics of patients, such as whether they have received chemotherapy or immune checkpoint inhibitors. Moreover, it remains unknown whether a regain of CD30 expression occurs in patients with previous reduced or loss of CD30 expression after BV therapy since the follow-up time is relatively short.

The specific mechanisms underlying CD30 downregulation remain unclear, which may include reduced CD30 transcription and cleavage of CD30 by a disintegrin and metalloproteinase 10 (ADAM10) [110,111,112]. CD30 shedding by ADAM10 activity decreases the binding sites for BV and generates competitive soluble CD30 to bind BV, thereby impairing the efficacy of BV [111,113]. Additionally, EVs released by CD30-positive cells also contain ADAM10. It gradually cleaves CD30 within the EVs, limiting the crossfire functionality of the EVs to present additional membrane-associated CD30 sites for BV on bystander cells [85,113]. Selective inhibitors of ADAM10 maintained the expression of CD30 on the cell surface and enhanced the efficacy of BV in a 3D model in vitro [114]. A previous study also found that IL-4 downregulated the expression of CD30 in neoplastic canine mast cells, and pre-incubation with IL-4 decreased the responsiveness of these cells to BV treatment [115]. SLC39A7, a zinc transporter in the ER, regulated the translocation of CD30 from the ER to the cell surface by modulating intracellular zinc homeostasis and played an important role in the response to BV [65]. However, it is unclear whether IL-4 or SLC39A7 is abnormal in expression or function in TCL patients.

Collectively, these results suggest that the expression levels of membranous CD30 may influence patients’ responses to BV treatment, while further studies are needed to verify this hypothesis with standardized and uniform methods for detecting CD30 expression in large cohorts.

### 5.2. Increased Drug Efflux by MDR1

Another reported mechanism is the resistance toward MMAE through upregulation of the multidrug resistance protein 1 (MDR1). However, such an effect was found in a cHL-resistant cell line but not in ALCL-resistant cell lines [110]. MDR1, a member of the ATP-binding cassette transporter protein family, plays a crucial role in exporting numerous exogenous compounds from cells, serving as a common mechanism of resistance to many ADCs [77]. Inhibition of MDR1 expression by cyclosporine A or elacridar can increase the intracellular MMAE level and efficacy of BV in cHL cell lines and R/R HL patients [116,117]. Wei et al. [118] revealed that the increased expression of MDR1 resulted from the activation of NF-kB pathways in tumor cells, and the IKKβ inhibitor MLN120B could restore their sensitivity to BV. Ubiquitin-editing enzyme A20 and RBX1 have been identified as key regulators that inhibit the expression of MDR1. Knocking down these molecules significantly decreased the sensitivity of tumor cells to BV [118]. In summary, MDR1 is a promising target for alleviating drug resistance in cHL, but its role in TCL needs further investigation.

## 6. Conclusions

The successful development and application of BV have changed the management for TCL. Recent advances in clinical studies highlight the benefits of combining BV with other therapies, especially chemotherapy. A prime example of this is the ECHELON-2 trial, a randomized phase III trial in PTCL patients. It demonstrated a better 5-year OS for BV + CHP compared to CHOP (75.1% vs. 61.0%, HR = 0.72) and led to the approval of BV + CHP as a front-line therapy for CD30-positive PTCL, which significantly influenced clinical practice. More ongoing studies are exploring the combination potential of BV with other drugs. In this review, we have synthesized these clinical trials and offered a holistic perspective on the current treatment landscape of BV combination therapies. Notably, this review is also the first to discuss the action and resistance mechanisms of BV comprehensively. The evolving understanding of how BV modulates anticancer responses, including its effects on both tumors and the tumor microenvironment, supports its encouraging efficacy in clinical applications. However, studies have also raised some contradictions and unresolved issues, including the predictive value of CD30 in treatment outcomes and the existence of non-responders. New insights into the mechanisms of resistance will help address these challenges and ultimately provide more promising therapeutic options for TCL patients.

## Figures and Tables

**Table 1:** CD30 expression levels in main entities of PTCL and CTCL.

Subtype	CD30 Expression Level (%)	Citation
PTCL		
PTCL-not otherwise specified	32–64 *	[30,40,41]
sALCL	100 *	[30,42]
ATLL	55–68 *	[30,43]
AITL	49–76 *	[30,40,44]
EATL	50–100 *	[30,40,44]
ENKTL	46–80 *	[30,40,45,46]
CTCL		
MF/SS	59 *	[40,47]
Transformed MF	48–100 *	[40,48,49]
pcALCL	>75 (required for diagnosis) ^#^	[50]
LyP	100 (variable in Type B) ^#^	[50]

* Percentages of patients with CD30-positivity determined via immunohistochemistry. ^#^ Percentages of CD30^+^ cells in lymphoma tissue assessed via immunohistochemistry. PTCL, peripheral T-cell lymphoma; sALCL, systemic anaplastic large cell lymphoma; ATLL, HTLV-1-associated adult T-cell leukemia/lymphoma; AITL, angioimmunoblastic T-cell lymphoma; EATL, enteropathy-associated T-cell lymphoma; ENKTL, extra-nodal NK/T-cell lymphoma; MF, mycosis fungoides; SS, Sézary syndrome; pcALCL, primary cutaneous anaplastic large cell lymphoma; LyP, lymphomatoid papulosis.
